# Treatment of Stomatitis

**Published:** 1887-11

**Authors:** 


### Treatment of Stomatitis.

EDITORS MED. AND SUR. REPORTER,

*Sirs*:—1. Will you, or some reader of the REPORTER, furnish formulas, through the REPORTER, for canker, or sore mouth in infants?

2. Will you state if sore mouth is ever caused by the effect of mercury in chalk mixture? J. E. STILES.

Red Lake Falls, Minn., June 27, 1887.

[In reply to question 1, we would suggest the following line of treatment:

For local and general antiseptic effect:

℞ Potas. chlorat.................................... ʒj

Aquæ destillat.................................... f℥ij

Syr. simplicis.................................... f℥j

M. Sig.—Use as a wash for mouth and give a teaspoonful every four hours.

Or,

℞. Acid. boric.................................... ʒij

Infus. med. sassaf.................................... f℥ij

Glycerinæ.................................... f℥j

M. Sig.—Use as a wash for the mouth and give a teaspoonful every four hours.

To regulate the bowels:

℞. Ol. ricini....................................
Syr. rhei. aromat.................................... aa f℥j

M. Sig.—Give one or two teaspoonfuls, according to age.

As a tonic:

℞. Ferri. et potas. tart.................................... gr. xxiv

Aquæ bullient.................................... f℥j

Syr. zingiber.................................... f℥ss

M. Sig.—Give a teaspoonful t. d....................

In reply to question 2, we would say that chalk mixture contains no mercury; if mercury were *added to it*, it would be very likely to produce a constitutional effect, perhaps a stomatitis.—EDITORS OF THE REPORTER.]

